# The Detection of Acute Risk of Self-injury Project: Protocol for an Ecological Momentary Assessment Study Among Individuals Seeking Treatment

**DOI:** 10.2196/46244

**Published:** 2023-06-15

**Authors:** Glenn Kiekens, Laurence Claes, Steffie Schoefs, Nian D F Kemme, Koen Luyckx, Evan M Kleiman, Matthew K Nock, Inez Myin-Germeys

**Affiliations:** 1 Faculty of Psychology and Educational Sciences KU Leuven Leuven Belgium; 2 Center for Contextual Psychiatry KU Leuven Leuven Belgium; 3 Faculty of Medicine and Health Sciences University of Antwerp Antwerp Belgium; 4 University of the Free State Bloemfontein South Africa; 5 Rutgers The State University of New Jersey New Jersey, NJ United States; 6 Department of Psychology Harvard University Cambridge, MA United States

**Keywords:** nonsuicidal self-injury, suicidal thoughts and behaviors, real-time, experience sampling, ecological momentary assessment, digital interventions, mobile phone

## Abstract

**Background:**

Nonsuicidal self-injury (NSSI) is a major mental health concern. Despite increased research efforts on establishing the prevalence and correlates of the presence and severity of NSSI, we still lack basic knowledge of the course, predictors, and relationship of NSSI with other self-damaging behaviors in daily life. Such information will be helpful for better informing mental health professionals and allocating treatment resources. The DAILY (Detection of Acute rIsk of seLf-injurY) project will address these gaps among individuals seeking treatment.

**Objective:**

This protocol paper presents the DAILY project’s aims, design, and materials used. The primary objectives are to advance understanding of (1) the short-term course and contexts of elevated risk for NSSI thoughts, urges, and behavior; (2) the transition from NSSI thoughts and urges to NSSI behavior; and (3) the association of NSSI with disordered eating, substance use, and suicidal thoughts and behaviors. A secondary aim is to evaluate the perspectives of individuals seeking treatment and mental health professionals regarding the feasibility, scope, and utility of digital self-monitoring and interventions that target NSSI in daily life.

**Methods:**

The DAILY project is funded by the Research Foundation Flanders (Belgium). Data collection involves 3 phases: a baseline assessment (phase 1), 28 days of ecological momentary assessment (EMA) followed by a clinical session and feedback survey (phase 2), and 2 follow-up surveys and an optional interview (phase 3). The EMA protocol consists of regular EMA surveys (6 times per day), additional burst EMA surveys spaced at a higher frequency when experiencing intense NSSI urges (3 surveys within 30 minutes), and event registrations of NSSI behavior. The primary outcomes are NSSI thoughts, NSSI urges, self-efficacy to resist NSSI, and NSSI behavior, with disordered eating (restrictive eating, binge eating, and purging), substance use (binge drinking and smoking cannabis), and suicidal thoughts and behaviors surveyed as secondary outcomes. The assessed predictors include emotions, cognitions, contextual information, and social appraisals.

**Results:**

We will recruit approximately 120 individuals seeking treatment aged 15 to 39 years from mental health services across the Flanders region of Belgium. Recruitment began in June 2021 and data collection is anticipated to conclude in August 2023.

**Conclusions:**

The findings of the DAILY project will provide a detailed characterization of the short-term course and patterns of risk for NSSI and advance understanding of how, why, and when NSSI and other self-damaging behaviors unfold among individuals seeking treatment. This will inform clinical practice and provide the scientific building blocks for novel intervention approaches outside of the therapy room that support people who self-injure in real time.

**International Registered Report Identifier (IRRID):**

DERR1-10.2196/46244

## Introduction

### Background

Nonsuicidal self-injury (NSSI), the direct and deliberate damage of one’s body tissue without suicidal intent (eg, cutting and hitting oneself), is a major mental health concern worldwide. Epidemiological surveys indicate that 1 in 5 individuals engage in NSSI before the age of 25 years [1-3], with 12-month rates in the 8% to 19% range [1,2] and many individuals reporting persistent NSSI for several years [4]. These rates are considerably higher among individuals seeking treatment [5-7], with more than half of adolescent inpatients and 1 in 10 emerging adult and adult outpatients reporting monthly NSSI [8,9]. Furthermore, the presence of NSSI predicts rehospitalization [10], is highly stigmatized [11], and is uniquely associated with an increased risk for psychiatric disorders [2,9,12-15] and suicidal thoughts and behaviors (STB). For instance, research shows that people who engage in NSSI are more likely to make a suicide attempt independent of mental disorders [16,17], with those engaging in repetitive persistent NSSI being most likely to attempt suicide and experience adverse psychosocial outcomes [18]. As such, there is an urgent need to better understand, predict, and prevent self-injury among people who frequently engage in these behaviors.

Unfortunately, we still lack basic knowledge of the descriptive nature of NSSI and how, why, and when self-injury unfolds in everyday life [19], a prerequisite for risk screening and intervention to prevent NSSI and clinically associated outcomes (eg, suicide attempts). This lack of progress is largely because most empirical studies have focused on establishing the prevalence and correlates of the presence and severity of NSSI using cross-sectional designs. Although longitudinal studies have been conducted, they typically used observation windows from months to years [19-21], providing a long-distance view of who is at greater risk of engaging in NSSI compared with others. However, such a nomothetic approach does not clarify *when* someone is at risk in the short term and in what daily life contexts they are more at risk and will thus not help clinicians to decide whether an individual is likely to self-injure within the next days and weeks. Making these decisions requires an idiographic approach and knowledge of the course, contexts of risk, and the relationship of NSSI with other self-damaging behaviors in daily life. Such information would be helpful for better informing mental health professionals and allocating treatment resources.

Similar to other frequently co-occurring self-damaging behaviors (eg, attempting suicide, binge eating, purging, and binge drinking) [2,13,22], NSSI cannot be ethically induced in the laboratory but occurs in people’s everyday lives. Although studying NSSI as it unfolds in real time has historically been challenging, recent technological advancements have made it possible to do exactly this, which has the potential to substantially improve our understanding and inform prevention efforts [19,23]. Ecological momentary assessment (EMA; also called experience sampling, ambulatory assessment, or real-time monitoring) involves prompting individuals multiple times per day via their mobile phone to provide in-the-moment information on social appraisals, emotions, cognitions, and behavioral patterns as they are lived in the flow of daily life [24-26]. Prior research suggests that NSSI thoughts can change substantially across hours [27], essentially necessitating EMA to (1) capture these real-time changes and (2) enable the identification of factors that predict these changes in daily life.

**Figure 1:**
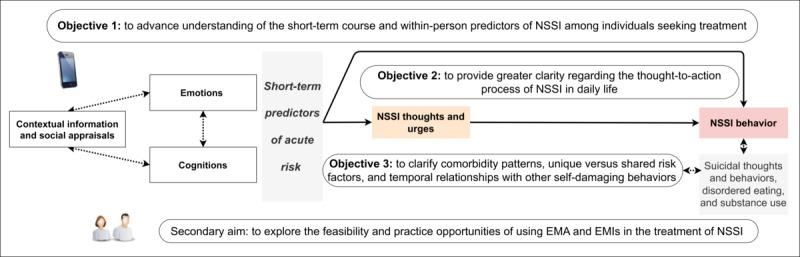
Graphical overview of the study objectives. EMA: ecological momentary assessment; EMI: ecological momentary intervention; NSSI: nonsuicidal self-injury.

Advancing insight into the course and contexts of elevated risk for NSSI thoughts (ie, *thinking about deliberately hurting oneself*), NSSI urges (ie, *a difficult-to-control desire to self-injure*), self-efficacy to resist NSSI (ie, *confidence in one’s ability not to engage in self-injury*), and NSSI behavior (ie, *engagement in self-injury*) would uniquely inform researchers and clinicians about how self-injury unfolds and is experienced by those at highest risk. This *in-the-moment* information can transform our ability to prevent and manage NSSI among individuals seeking treatment by providing the scientific building blocks for novel digital interventions that support people who self-injure in real time [19,28,29]. The DAILY (Detection of Acute rIsk of seLf-injurY) project aims to address these gaps using a 28-day EMA protocol among individuals seeking treatment. This protocol paper presents the project’s aims, design, and materials used. Figure 1 provides a graphical presentation of the primary objectives of the DAILY project.

### Objectives

The first primary objective is to clarify the short-term course and within-person predictors of NSSI. Initial work among community samples suggests that NSSI thoughts are usually short-lived and moderate in intensity [30-32], with a higher risk of more intense and persistent NSSI thoughts toward the evening [32] and an average of 1.6 (SD 1.1) NSSI behaviors per week [30]. Surprisingly, however, the short-term clinical course of NSSI remains to be comprehensively investigated among individuals seeking treatment. The intensive nature of the longitudinal data we collect will allow for a detailed picture of the thought and behavioral patterns and help us to address several critical unanswered questions about the course of NSSI among individuals seeking treatment: how much do NSSI thoughts, NSSI urges, self-efficacy to resist NSSI, and NSSI behavior (1) vary between people seeking treatment, (2) vary within people seeking treatment, (3) vary throughout the day and the week, and (4) covary and predict each other prospectively in daily life?

Guided by contemporaneous models of NSSI [33-35] and emerging work [36-38], we will evaluate contextual information, social appraisals, emotions, and cognitions as within-person risk and protective factors for cognitive and behavioral NSSI outcomes. We hypothesize that the risk of NSSI behavior will be higher when people are alone [30], after situations characterized by negative social appraisals and perceived conflict [39,40], and after increased negative and decreased positive affect [41]. Given that specific emotions may incrementally predict higher risk (eg, feeling stressed) [30,42] or protect more strongly against NSSI (eg, feeling relaxed) [27], the findings will also be analyzed using specific emotions as units of analysis. On the basis of the benefits and barriers model of NSSI [34,43], which argues for a unique role of self-critical thoughts in explaining NSSI behavior, we will evaluate the association between momentary fluctuations in self-critical thinking and NSSI urges and NSSI behavior. Building upon the emotional cascade model [35,44], which posits that reinforcing cyclic cascades between rumination and negative affect form a dynamic process in which self-injury is suggested to break this cycle by distracting from rumination, the utility of momentary ruminative thinking and the interplay with negative emotions in predicting NSSI behavior will be investigated. However, because the relevance of these theoretically inspired within-person risk factors for NSSI might differ between individuals [45,46], we will also determine variability in individual risk associations.

A second primary objective is to advance understanding of the transition from NSSI thoughts and urges to NSSI behavior. Although existing theoretical models of NSSI do not distinguish between NSSI thoughts or urges and NSSI behavior, contemporary “ideation-to-action” theories of suicide explicitly make this distinction [47] and suggest that the factors that lead to suicidal thoughts or urges are not necessarily the same factors that lead to behavior (ie, suicide attempts). Emerging evidence indicates that incorporating ideation-to-action thinking might be equally relevant for NSSI. For instance, preliminary work suggests that previously identified risk factors for NSSI behavior (eg, emotional factors) might uniquely predict NSSI thoughts but not the occurrence of NSSI behavior [27]. Inspired by the cognitive-emotional model of NSSI [33], we anticipate that momentary self-efficacy to resist NSSI will be a potent protective factor against NSSI behavior. People retrospectively report that the transition between NSSI thoughts or urges and NSSI behavior is typically <30 minutes [30,31], meaning that there might be a brief window of opportunity to intervene during these moments of acute risk for NSSI behavior. Therefore, in an attempt to inform and facilitate such intervention efforts, we will make a thought-to-action distinction and clarify the specificity of risk and protective factors to identify factors that increase or decrease the likelihood of transitioning from NSSI thoughts or urges to NSSI behavior in real time among individuals seeking treatment.

A third primary objective is to provide novel data on the co-occurrence of NSSI with other self-damaging behaviors in daily life that may represent different forms of behavior that serve the same function (ie, disordered eating, substance use, and STB) [13,30,48-51]. For example, previous daily diary studies found that 42% to 53% of the adolescents and emerging adults who engage in NSSI reported at least 1 episode of disordered eating and suicidal ideation [40,52], with suicidal thoughts being present on most days when adolescents engaged in NSSI [53]. Similarly, Nock et al [30] observed that NSSI thoughts co-occurred 13% to 18% of the time with thoughts of substance use and disordered eating. Therefore, disordered eating (ie, restrictive eating, binge eating, and purging), substance use (ie, binge drinking and smoking cannabis), and STB will be assessed as secondary outcomes. From a transdiagnostic approach, this will help to clarify comorbidity patterns, unique versus shared risk factors, and temporal relationships with other self-damaging behaviors in daily life [13,19,54]. In addition, it will allow us to address whether the dynamic characteristics of NSSI thoughts or urges (ie, an individual’s within-person average and variability) and frequency of NSSI behavior—as observed in the EMA—predict changes in NSSI trajectories and the presence of comorbid self-damaging behaviors among individuals seeking treatment [55]. For instance, it may be that persistently high NSSI urges and frequent NSSI behavior increase the risk of STB [53]. Alternatively, there could also be a behavior shift such that a reduced frequency of NSSI leads some individuals to take up another behavior that serves a similar function.

Finally, a secondary aim is to evaluate the perspectives of individuals seeking treatment and mental health professionals regarding the feasibility, scope, and utility of digital self-monitoring and novel interventions. There is a growing awareness that the clinical implementation of EMA and ecological momentary interventions (EMIs), which use EMA to deliver support in real-time, provides new opportunities to make individuals more actively involved in their treatment and to better match treatments to their needs [19,24,56,57]. For instance, EMA could facilitate self-insight about relevant processes [19,58,59], whereas EMIs would allow for providing support outside of the therapy room in daily life. Initial findings indicate the acceptability and potential of EMIs for NSSI and suicide prevention [29,60,61], but, despite the clear clinical potential, this remains a largely underexplored area. Importantly, however, the development of EMIs will advance more rapidly and be more easily clinically implemented when a user-centered design is employed. Such a design actively involves and acknowledges end users from the initial stage of development to understand the goals, challenges, and motivations for future EMIs that seek to facilitate young people’s recovery from self-injury and other self-damaging behaviors [62].

## Methods

### Target Population and Recruitment

To investigate the objectives of the DAILY project, the target population is adolescents (aged 15-18 years), emerging adults (aged 19-29 years), and adults (aged 30-39 years) seeking treatment and their mental health professionals. They are recruited from mental health services across the Flanders region of Belgium, including 9 inpatient services, 8 outpatient services, and 4 services with a hybrid care model. Of these services, 11 focus on emotion dysregulation and mood disorders, 5 focus on social-emotional difficulties in transitioning from adolescence to emerging adulthood, 3 focus on eating disorders, and 2 are private practices. Potential eligible participants are informed about the study via mental health professionals, study flyers, and information moments at these mental health services. The inclusion criteria for patients are as follows: (1) being aged between 15 and 39 years, (2) having sufficient Dutch-language proficiency, (3) past-month NSSI thoughts/behaviors, and (4) receiving inpatient or outpatient treatment. Individuals with cognitive deficits that preclude comprehension of materials are excluded from participation. Although adult participants (aged ≥18 years) could opt to continue participation when they decided to stop treatment, a treating mental health professional had to be involved for minors (<18 years) during the entire monitoring period, meaning that participation automatically ended when they interrupted their treatment before the end of the 28-day EMA protocol. The inclusion criterion for mental health professionals is being a licensed psychiatrist, clinical psychologist, or mental health nurse.

### Ethics Approval

All phases of the DAILY project were approved by the Ethics Committee Research UZ/KU Leuven on February 10, 2021 (s64989 and B3222020000343), and all procedures align with the 1964 Helsinki declaration and its later amendments.

### Procedure and Protocol

**Figure 2:**
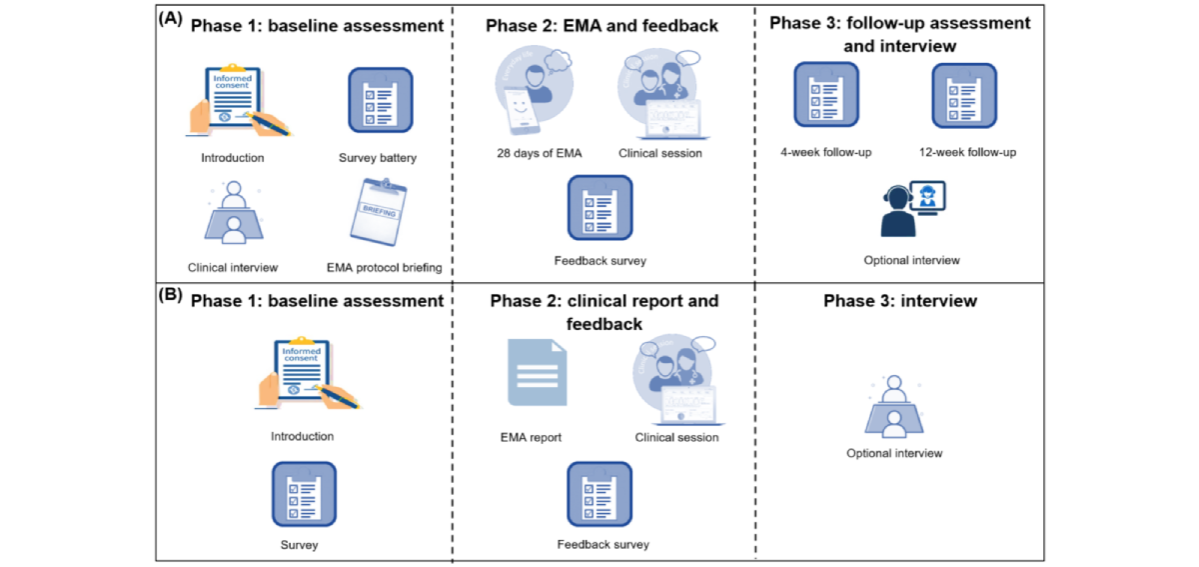
Graphical overview of the study procedures for (A) individuals seeking treatment and (B) mental health professionals. EMA: ecological momentary assessment.

The DAILY project consists of 3 phases, including a baseline assessment (phase 1), 28 days of EMA followed by a clinical session and feedback survey (phase 2), and 2 follow-up surveys and an optional interview (phase 3). Figure 2 provides a graphical overview of the project.

#### Phase 1: In-Person Baseline Assessment of Individuals Seeking Treatment

In the first phase, a psychologist meets with the patient (hereinafter referred to as the participant) to give them detailed information, conduct the baseline assessment, and provide training on completing the EMA protocol. The informed consent process informs participants about the study demands and goals, the safety protocol, reimbursement scheme, the researchers’ responsibility concerning risk monitoring, and the implications of this responsibility (ie, when the duty of care overrides the confidentiality principle). All participants provide written informed consent or assent. In addition, parents or legal caregivers provide written informed consent for minors aged 15 to 17 years. The baseline assessment consists of a survey battery and a clinical interview. Table 1 presents the constructs covered in the survey battery assessed via REDCap (Research Electronic Data Capture; Vanderbilt University) [63]. The clinical interview assesses the participants’ history of NSSI and STB using the well-validated Self-Injurious Thoughts and Behaviors Interview [64,65]. In addition, the Structured Clinical Interview for DSM-5 [66], is used to assess common comorbid mental disorders, including major depressive disorder, alcohol use disorder, substance use disorder, panic disorder, generalized anxiety disorder, posttraumatic stress disorder, and eating disorders [2,15,22]. Interrater reliability will be examined within a subset of 20% of the diagnostic interviews (approximately 24 participants) by calculating the percentage of agreement and Cohen κ coefficients for overall diagnosis [67]. During the clinical interview, participants are asked about personal strategies that they consider helpful to resist engaging in NSSI and other self-damaging behaviors (see *Phase 2: Safety Measures and Risk Monitoring* section).

Phase 1 concludes with an orientation and training on completing the EMA protocol via m-Path, a user-friendly and secure smartphone app and platform created by researchers at KU Leuven that is General Data Protection Regulation (GDPR)–compliant for real-time and real-world data collection [68]. We assign all participants a study alias to ensure that no identifying information is shared with the software platform. All communication between participants’ smartphones and the server is end-to-end encrypted and securely uploaded after each interaction [68]. Participants without a smartphone are loaned a device with a prepaid data plan for data collection. Study staff help participants to download the app on their phone (Android or IOS) or the loaned device (Android), discuss the EMA protocol’s content and procedures, answer participants’ questions, and initiate a practice EMA survey for the participant. Phase 1 takes approximately 3 to 4 hours to complete.

**Table 1:** Overview of the variables assessed in the baseline survey battery and clinical interview.

Baseline assessment			Measure	Source		Items, n; response scale
**Survey battery**						
	Sociodemographic information	Age, gender, education, profession, and sexual orientation		Items from Kiekens et al [2] and self-developed	6; mixed	
	Trait negative and positive affect	Positive and Negative Affect Schedule		Original [69] and Dutch translation [70]	20; 5-point Likert scale	
	Trait rumination	Ruminative Response Scale		Original [71] and Dutch translation [72]	22; 4-point Likert scale	
	Trait perseverative thinking	Perseverative Thinking Questionnaire		Original [73] and Dutch translation [74]	15; 5-point Likert scale	
	Trait self-criticism and self-reassurance	Forms of Self-Criticizing or Attacking and Self-Reassuring Scale		Original [75] and Dutch translation [76]	22; 5-point Likert scale	
	Trait emotion dysregulation	Difficulties in Emotion Regulation Scale-Short Form		Original [77] and Dutch translation [78]	18; 5-point Likert scale	
	Trait negative and positive urgency	UPPS-P^a^ Impulsive Behavior Scale		Original [79] and Dutch translation [80]	26; 4-point Likert scale	
	Identity	Identity scale from the Erikson Psychosocial Stage Inventory		Original [81] and Dutch translation [82]	12; 5-point Likert scale	
	Depression, anxiety, and stress^b^	Depression, anxiety, and stress scales (past week)		Original [83] and Dutch translation [84]	21; 4-point Likert scale	
	NSSI^c^ expectancies	Nonsuicidal Self-Injury Expectancy Questionnaire		Original [85] and Dutch translation: FBT^d^	25; 5-point Likert scale	
	Self-efficacy to resist NSSI in the next weeks and across contexts	Adapted Self‑Efficacy to Avoid Suicide Action Scale; Self‑Efficacy to Resist Nonsuicidal Self‑Injury Scale		Original [86] and Dutch translation [27]; Original [87], and Dutch translation: FBT	6; 10-point scale; 24; 4-point Likert scale	
	Disordered eating behaviors and attitudes^b^	Eating Disorder Examination Questionnaire (version 6.0)		Original [88] and Dutch translation [89]	30; mixed	
	Positive body image	Body Appreciation Scale-2		Original [90] and Dutch translation [91]	10; 5-point Likert scale	
	Expectancies about eating	Subscales of the Eating Expectancy Inventory		Original [92] and Dutch translation [93]	28; 7-point Likert scale	
	Eating and body image self-efficacy	Eating Disorder Recovery Self-Efficacy Questionnaire		Original [94] and Dutch translation: FBT	23; 7-point Likert scale	
	Borderline personality disorder	McLean Screening Instrument for Borderline Personality Disorder		Original [95] and Dutch translation [96]	10; dichotomous scale	
	Damaging and impulsive behaviors^b^	Self-developed assessment of 13 behaviors		N/A^e^	13-26; mixed	
	Self-efficacy to resist drinking	Drinking Refusal Self-Efficacy Questionnaire-Revised		Original [97] and Dutch translation [98]	19; 6-point Likert scale	
	General self-efficacy	General Self-Efficacy Scale		Original [99] and Dutch translation [100]	10; 4-point Likert scale	
	Perceived social support	Multidimensional Scale of Perceived Social Support		Original [101] and Dutch translation [102]	16; 5-point Likert scale	
	Rejection	Perceived Rejection Scale of Adult Toolbox Social Relationship Scales		Original [103] and Dutch translation: FBT	8; 5-point Likert scale	
	Affinity with technology	Adapted Affinity for Technology Scale		Original [104] and Dutch translation: FBT	10; 7-point Likert scale	
**Clinical interview**						
	NSSI thoughts, NSSI behaviors, DSM-5^f^ NSSI disorder, suicide ideation, suicide plan, suicide attempt, age of onset, frequency, recency, functions of NSSI, experience of pain during NSSI, and impact of NSSI^b^	Adapted Self-Injurious Thoughts and Behaviors Interview		Original [64] and Dutch translation [22]	29-41; mixed	
	Self-efficacy to resist suicide^b^	Self‑Efficacy to Avoid Suicide Action Scale		Original [86] and Dutch translation [27]	6; 10-point scale	
	Major depressive disorder, alcohol use disorder, substance use disorder, panic disorder, generalized anxiety disorder, posttraumatic stress disorder, and eating disorders	Structured Clinical Interview for DSM-5		Original [66] and Dutch translation [105]	18-310; mixed	

^a^UPPS-P: urgency, premeditation, perseverance, sensation seeking, and positive urgency.

^b^Reassessed in the web-based follow-up surveys 4 and 12 weeks after the 28-day real-time monitoring period.

^c^NSSI: nonsuicidal self-injury.

^d^FBT: forward-backward translation.

^e^N/A: not applicable.

^f^DSM-5: Diagnostic and Statistical Manual of Mental Disorders, Fifth Edition.

#### Phase 1: Web-Based Baseline Assessment of Mental Health Professionals

Each mental health professional also provides informed consent the first time and completes a brief survey assessing sociodemographic (age and gender) and professional information (profession, level of education, and years of experience). In addition, they can provide the research team with 1 to 3 additional EMA questions when considered relevant for a specific individual.

#### Phase 2: EMA Sampling Design and Content

**Figure 3:**
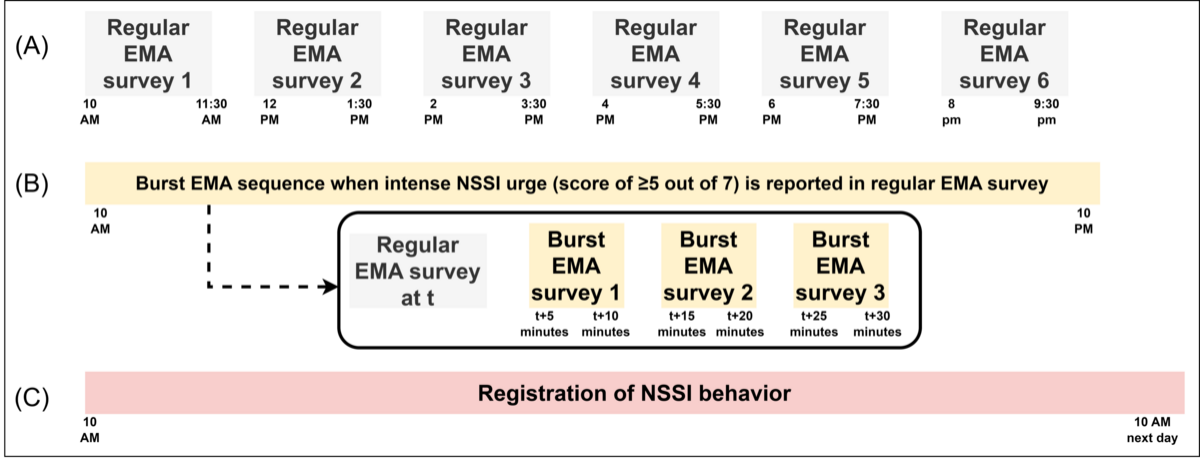
Graphical overview of the ecological momentary assessment (EMA) sampling schedule. (A) Six semirandom regular EMA surveys taken on average every 2 hours during waking hours between 10 AM and 9:30 PM. (B) Three burst EMA surveys spaced at a higher frequency of 5 to 10 minutes apart in the 30 minutes after intense nonsuicidal self-injury (NSSI) urges in the regular EMA surveys. (C) Event registrations via a push button to register the timing of NSSI behavio.

The second phase starts on the morning after the baseline assessment. It involves a 28-day EMA protocol that consists of (1) six semirandom regular EMA surveys administered on average every 2 hours during waking hours between 10 AM and 9:30 PM, (2) three burst EMA surveys spaced at a higher frequency of 5 to 10 minutes apart in the 30 minutes after intense NSSI urges are reported in the regular EMA surveys (score of ≥5 on a 7-point item), and (3) event registrations via a push button to record the timing of NSSI behavior. Figure 3 shows the sampling schedule of the DAILY project, which includes a minimum of 168 regular EMA surveys and a theoretical maximum of 672 EMA surveys (when a burst sequence is triggered during each regular survey). To ensure that we capture people in their ongoing activities and to avoid retrospective reporting, participants must register responses to the regular EMA within 15 minutes of receipt and to the burst EMA surveys within 5 minutes of receipt. In case of nonresponse to the regular EMA surveys, 1 reminder is sent after 10 minutes. After the first day and each week subsequently, a team member telephoned the participants to check whether everything is going well, provide feedback about study compliance, and respond to concerns or difficulties concerning the app and the self-monitoring.

Table 2 presents the EMA constructs and items in the DAILY project, including emotions and cognitions (block A); contextual information and social appraisals (block B); NSSI thoughts, NSSI urges, self-efficacy to resist NSSI, and NSSI behavior (block C); and the screening of other self-damaging behaviors and experienced momentary burden (block D, part 1). EMA items were as much as possible selected or modified from prior EMA studies and conventional survey questionnaires [27,86,90,106-108]. Questions are branched so that participants only have to complete relevant EMA items (eg, social appraisals are context specific). The order in which emotions and cognitions are presented is randomized within individuals across the EMA surveys (because these items are not conditional on each other). Specific emotions for negative and positive affect are selected because they represent all 4 quadrants of the affective circumplex defined by valence and arousal dimensions [109]. All continuous EMA items are assessed on 7-point rating scales ranging from not at all or absent to very much or very strong (Table 2). Although NSSI behavior is assessed retrospectively during each EMA survey (ie, “Since the last beep, have you deliberately hurt yourself without wanting to die?”), participants are instructed to register the timing of NSSI behavior through an event marker. This occurred on a wearable wireless device (Chill Band +; IMEC International) for the first 12 participants. However, owing to practical and technical issues with these devices, the remaining participants used an event registration push button in the m-Path app [68].

During each regular EMA survey, we also screened whether participants experienced thoughts, had an urge, or engaged in 6 other self-damaging behaviors, including restrictive eating, purging, binge eating, STB, binge drinking, and smoking cannabis (block D, part 1). If they answer ≥1 of these self-damaging behaviors affirmatively, additional items assess thoughts, urges, behavior engagement, and self-efficacy to resist the endorsed behavior(s) (block D, part 2, Table 3). Finally, participants rate the extent to which they consider an EMA survey burdensome. The regular EMA surveys include 28 to 31 items (excluding optional individual-specific questions), with 4 additional items for each of the other self-damaging behaviors endorsed on the screening item (Table 3). The burst EMA surveys include 21 to 22 items, assessing only emotions and cognitions (block A) and NSSI outcomes (block C).

**Table 2:** Ecological momentary assessment (EMA) constructs and items.

EMA constructs^a^			Items		Response category
**Block A: emotions and cognitions (17 items)**					
	Momentary negative affect	[Right now]: “I feel anxious,” “I feel stressed,” “I feel irritated,” “I feel sad,” and “I feel insecure”		7-point scale (ranging from not at all to very much)	
	Momentary positive affect	[Right now]: “I feel cheerful” and “I feel relaxed”		7-point scale (ranging from not at all to very much)	
	Momentary emptiness	[Right now]: “I feel empty”		7-point scale (ranging from not at all to very much)	
	Momentary loneliness	[Right now]: “I feel lonely”		7-point scale (ranging from not at all to very much)	
	Momentary emotion dysregulation	[Right now]: “My emotions are overwhelming me”		7-point scale (ranging from not at all to very much)	
	Momentary rumination	[Right now]: “I am repeatedly thinking about the same problem”		7-point scale (ranging from not at all to very much)	
	Momentary self-criticism	[Right now]: “I am disappointed in myself”		7-point scale (ranging from not at all to very much)	
	Momentary other-criticism	[Right now]: “I am disappointed in others”		7-point scale (ranging from not at all to very much)	
	Momentary body image	[Right now]: “I am satisfied with the way I look” and “I feel at ease in my body”		7-point scale (ranging from not at all to very much)	
	Momentary identity	[Right now]: “I doubt who I am” and “I know what I stand for”		7-point scale (ranging from not at all to very much)	
**Block B: contextual information and social appraisals (5-7 items)**					
	Momentary situational context	“Where are you?”		(a) At home; (b) at a friend’s or family’s place; (c) school or work; (d) car, train, bus; (e) other indoors; (f) other outdoors	
	Momentary activity	“What were you doing just before the beep?” (If g) “Please describe briefly what you are doing”		(a) Nothing, (b) work or studying, (c) household chores, (d) conversation, (e) leisure active (eg, sports), (f) leisure passive (eg, watching television), (g) something else	
	Momentary appraisal of activity	“I enjoy this activity”		7-point scale (ranging from not at all to very much)	
	Momentary social context	“Are you alone or with others?” (If b) “Who are you with physically?” (If c) “Who are you with online?”		(a) Alone, (b) others physically, (c) others on the web (a) Partner, (b) family, (c) friends, (d) colleagues, (e) acquaintances, (f) other patients, (g) unfamiliar people	
	Momentary appraisal of social context	(If a): “I like being alone” (If b): “I enjoy the company of these people” (If c): “I feel connected to those I am online in contact with”		7-point scale (ranging from not at all to very much) 7-point scale (ranging from not at all to very much) 7-point scale (ranging from not at all to very much)	
**Block C: NSSI^b^ (4-5 items)**					
	Retrospective thoughts	“Since the last beep, have you considered deliberately hurting yourself without wanting to die?”		7-point scale (ranging from not at all to very much)	
	Momentary urge	“Right now, how strong is the urge present to hurt yourself without wanting to die?”		7-point scale (ranging from absent to very strong)	
	Retrospective behavior	“Since the last beep, have you deliberately hurt yourself without wanting to die (for example, cut, scratched, or hit yourself)?”		(a) No, (b) yes	
	Retrospective behavior (method)	(If b): “How have you self-injured?”		(a) Cutting or carving, (b) scratching, (c) hitting, (d) burning, (e) biting, (f) head banging, (g) wound interfering, (h) other method (describe briefly)	
	Momentary self-efficacy to resist	“Right now, how confident are you that you can resist engaging in NSSI?”		7-point scale (ranging from not at all to very)	
**Block D (part 1): screen other self-damaging thoughts or behaviors and interference (2 items)**					
	Screen comorbid thoughts or behaviors	“Indicate all other behaviors you thought about or engaged in since the last beep, or for which you have an urge right now”		(a) Restrictive eating, (b) binge eating, (c) vomiting deliberately, (d) killing myself, (e) binge drinking, (f) using cannabis, (g) none of these	
	Interference	“This beep disturbed me.”		7-point scale (ranging from not at all to very much)	

^a^Blocks A to D are assessed in the regular EMA surveys. Blocks A and C are only assessed in the burst EMA surveys.

^b^NSSI: nonsuicidal self-injury.

**Table 3:** Ecological momentary assessment of other self-damaging behaviors^a^.

Block D (part 2): Other self-damaging behaviors and items				Response category
**Restrictive eating (4 items)**				
	**Retrospective thoughts**			
		Since the last beep, have you thought about eating less to control your weight?	7-point scale (ranging from not at all to very much)	
	**Momentary urge**			
		Right now, how strong is the urge to eat less to control your weight?	7-point scale (ranging from absent to very strong)	
	**Retrospective behavior**			
		Since the last beep, have you eaten less to control your weight?	(a) No, (b) yes	
	**Momentary self-efficacy to resist**			
		Right now, how confident are you that you can resist eating less to control your weight?	7-point scale (ranging from not at all to very)	
**Binge eating (4 items)**				
	**Retrospective thoughts**			
		Since the last beep, have you thought about eating an unusually large amount of food?	7-point scale (ranging from not at all to very much)	
	**Momentary urge**			
		Right now, how strong is the urge to eat an unusually large amount of food?	7-point scale (ranging from absent to very strong)	
	**Retrospective behavior**			
		Since the last beep, have you experienced a binge-eating episode?	(a) No, (b) yes	
	**Momentary self-efficacy to resist**			
		Right now, how confident are you that you can resist eating an unusually large amount of food?	7-point scale (ranging from not at all to very)	
**Purging (4 items)**				
	**Retrospective thoughts**			
		Since the last beep, have you thought about vomiting deliberately?	7-point scale (ranging from not at all to very much)	
	**Momentary urge**			
		Right now, how strong is the urge to vomit deliberately?	7-point scale (ranging from absent to very strong)	
	**Retrospective behavior**			
		Since the last beep, have you vomited deliberately?	(a) No, (b) yes	
	**Momentary self-efficacy to resist**			
		Right now, how confident are you that you can resist vomiting deliberately?	7-point scale (ranging from not at all to very)	
**Suicidality (4 items)**				
	**Retrospective thoughts**			
		Since the last beep, have you thought about killing yourself?	7-point scale (ranging from not at all to very much)	
	**Momentary urge**			
		Right now, how strong is the urge to kill yourself?	7-point scale (ranging from absent to very strong)	
	**Retrospective behavior**			
		Since the last beep, have you made a suicide attempt?	(a) No, (b) yes	
	**Momentary self-efficacy to resist**			
		Right now, how confident are you that you can resist attempting suicide?	7-point scale (ranging from not at all to very)	
**Binge drinking (4 items)**				
	**Retrospective thoughts**			
		Since the last beep, have you thought about consuming an unusually large amount of alcohol?	7-point scale (ranging from not at all to very much)	
	**Momentary urge**			
		Right now, how strong is the urge to consume an unusually large amount of alcohol?	7-point scale (ranging from absent to very strong)	
	**Retrospective behavior**			
		Since the last beep, have you consumed an unusually large amount of alcohol?	(a) No, (b) yes	
	**Momentary self-efficacy to resist**			
		Right now, how confident are you that you can resist consuming an unusually large amount of alcohol?	7-point scale (ranging from not at all to very)	
**Cannabis use (4 items)**				
	**Retrospective thoughts**			
		Since the last beep, have you thought about smoking cannabis?	7-point scale (ranging from not at all to very much)	
	**Momentary urge**			
		Right now, how strong is the urge to smoke cannabis?	7-point scale (ranging from absent to very strong)	
	**Retrospective behavior**			
		Since the last beep, have you smoked cannabis?	(a) No, (b) yes	
	**Momentary self-efficacy to resist**			
		Right now, how confident are you that you can resist smoking cannabis?	7-point scale (ranging from not at all to very)	

^a^These other self-damaging behaviors are only assessed when screened positively in block D (part 1; Table 2) in the regular ecological momentary assessment surveys.

#### Phase 2: Safety Measures and Risk Monitoring

Participants are provided a 1-page information sheet with the contact information of the research team (study-specific telephone number and email address) as well as suicide and crisis hotlines during the baseline enrollment (phase 1). Although research suggests no reactivity or iatrogenic effects of repeated questioning about self-injurious thoughts and behaviors [110-112], there should be a proper safety protocol that not only matches participants’ needs [113] but also does not inadvertently defeat the study’s observational purpose [19,114]. Therefore, several measures are in place to support and safeguard participants’ safety during the EMA period. First, an automatic pop-up screen with resources is shown at the end of a regular EMA survey whenever a participant indicates an intense urge for NSSI or any other self-damaging behavior (response 6 or 7 on a 7-point scale). This pop-up item contains either standard resources that signpost participants to relevant support organizations, family and friends, and their mental health professional or the personal resources provided by the participant during phase 1 (randomized within individuals across surveys with an equal probability of 0.5).

Second, a safety protocol is activated whenever a participant reports having attempted suicide or is at imminent risk for attempting suicide, operationalized as having an intense urge to attempt suicide (response 6 or 7 on a 7-point scale) combined with momentary low self-efficacy to resist this suicidal urge (responses 1-3 on a 7-point scale). A second automatic pop-up screen is triggered whenever this response pattern occurs, encouraging participants to provide more in-the-moment information within an open text field. After submitting this item (even when left blank), this response pattern issues an alert to the research team (ie, flagged email to the study account that is consistently monitored during recruitment hours). This information is then shared via telephone with clinical staff members on duty at the care facility for inpatients, after which they take appropriate action. The participating mental health professional also receives an email with this information. When the safety alert concerns an outpatient, a licensed clinical psychologist from the research team conducts the risk assessment by telephone. For minors, the parents or legal guardians are also contacted by telephone when a minor participant cannot be reached or when the clinical psychologist of the research team considers this necessary after a risk assessment.

#### Phase 2: Feedback Report, Clinical Session, and Feedback Surveys

After the EMA period, a feedback report is shared with the mental health professional to be discussed during a regular therapy session. The report contains person-specific data on (1) general psychological functioning (ie, emotions and cognitions) and contextual information (eg, distribution of time alone vs with others face-to-face or on the web and activities in daily life); (2) the course of NSSI thoughts, NSSI urges, self-efficacy to resist NSSI, and NSSI behavior; and (3) the occurrence and intensity of other self-damaging thoughts and behaviors during the 28-day monitoring period. The section on general functioning includes pie charts displaying the proportion of time that someone spends across different contexts and box plots showing the median and variability of emotional and cognitive states. The section on NSSI provides information on the distribution and single-day average of NSSI thoughts, urges, and self-efficacy to resist NSSI (box plot and time series graphs). For example, high-risk days are operationalized as days on which the single-day average urge of NSSI is high (ie, mean response between 5-7 on a 7-point scale), coupled with low single-day average self-efficacy to resist NSSI (ie, mean response between 1-3 on a 7-point scale). In addition, the frequency of NSSI behaviors and types of methods are described, and NSSI urges are visualized across different situational, activity, and social contexts (bar charts). We also provide information about the occurrence of other self-damaging behaviors during the 28-day monitoring period. If the enrolled mental health professional cannot discuss the EMA feedback report (eg, because of treatment dropout or organizational issues), the patient can decide to have this session with another mental health professional or a clinical psychologist from the research team.

After the clinical session, an email is sent containing a link to a survey that assesses patients’ and mental health professionals’ experience of EMA and their perspectives on the EMA feedback report (Table 4). In case of nonresponse, 2 weekly reminders are sent. At this initial stage, we explore intrapersonal (ie, self-reflection and insight, self-efficacy, and subjective experiences) and interpersonal changes (ie, working alliance and helpful aspects during the clinical session) associated with the experience of EMA and the use of the EMA feedback report in treatment. The feedback survey also includes open questions assessing attitudes about self-monitoring, the content of the items, the feasibility of EMA, and the potential of EMIs for NSSI and other self-damaging behaviors (Table 4).

**Table 4:** Overview of constructs assessed in the feedback survey.

Survey battery	Measure and example item	Source	Items, n; response scale
Self-insight and reflection in general^a^	Adapted Self-Reflection and Insight Scale: “The self-monitoring made me more aware of my feelings and thoughts”	Original [116] and Dutch translation: FBT^b^	8; 6-point Likert scale
Self-efficacy in general	Adapted Self-Efficacy Scale: “By what I learned about myself through the self-monitoring, I feel that I am better able to solve difficult problems if I try hard enough”	Original [99] and Dutch translation [100]	10; 4-point Likert scale
Self-insight and reflection with respect to NSSI^c^ and other self-damaging behaviors^a^	Adapted Self-Reflection and Insight Scale: (1) experience of EMA^d^: “The self-monitoring made me more aware of my triggers for self-injury” and (2) use of EMA in therapy: “Discussing the monitoring results together with my clinician made me more aware of my triggers for self-injury”	Original [116] and Dutch translation: FBT	10; 6-point Likert scale
Self-efficacy resisting NSSI and other self-damaging behaviors	Adapted Self-Efficacy Scale: (1) experience of EMA: “By what I learned about myself through the self-monitoring, I now know better what to do when I am experiencing an urge to self-injure” and (2) use of EMA in therapy: “By discussing the monitoring results together with my clinician, I now know better what to do when I am experiencing an urge to self-injure”	Original [99] and Dutch translation [100]	10; 4-point Likert scale
Effect of discussing EMA results in therapy on the working alliance^a^	Adapted Working Alliance Inventory (short version): (1) task: “Discussing the results together helped us to understand which changes I need to make,” (2) bond: “I feel my clinician understands me better because of the monitoring results,” and (3) goal: “Discussing the results together makes me feel we are both working toward the same goals in therapy”	Original [117] and Dutch translation: FBT	12; 6-point Likert scale
Helpful aspects during clinical session^a^	Questions assessing any helpful or hindering aspects of the session and experience of clinician and therapeutic session as engaging and supportive, open and authentic, empathic, explorative, confrontational, and making positive progress: “I believe that my problems can be addressed through therapy”	Original [118] and Dutch translation [119]	40; mixed
Subjective experiences of EMA	Questions assessing (1) negative experience: “The self-monitoring questions caused me stress,” (2) positive experience: “I enjoyed the self-monitoring,” (3) learning experience: “I would describe the self-monitoring as a learning experience,” and (4) facilitates self-regulation: “The self-monitoring questions helped me to structure my thoughts”	Self-developed	12; 5-point Likert scale
Expectations of feedback and compensation	Questions assessing the overall and relative importance of receiving study findings, personal feedback, and financial compensation	Items from Kiekens et al [19] and self-developed	11; mixed
Qualitative feedback	Questions assessing attitudes about self-monitoring, content of the items, length of questionnaires, experience and feasibility of self-monitoring, utility and potential effect on the therapy process, and utility and scope of future interventions	Self-developed	10; open questions

^a^Clinician version also included these questions.

^b^FBT: forward-backward translation.

^c^NSSI: nonsuicidal self-injury.

^d^EMA: ecological momentary assessment.

#### Phase 3: Follow-up Surveys After 1 and 3 Months and (Optional) Interviews About Experiences and Attitudes Toward EMA and EMIs

Phase 3 includes 2 brief web-based follow-up surveys after 4 and 12 weeks and an optional interview. The surveys assess past-week psychological distress and the presence of NSSI and other self-damaging behaviors since completing the self-monitoring (first survey) or between the follow-up surveys (second survey; see questionnaires indicated with subscript in Table 1). In case of nonresponse, 2 weekly reminders are sent. The qualitative interviews assess more in depth the experiences of using EMA and the utility, content, and design of future EMIs for NSSI among a voluntary subsample of individuals seeking treatment (online after the feedback session) and mental health professionals (on site after they have participated with at least 2 patients in the study). The interview guides can be consulted on the project’s Open Science Framework (OSF) page [115].

##### Participant Incentives and Engagement

Participants are recognized as valued contributors to the research and receive financial incentives, information about the overall findings, and feedback on their data. Patients are financially compensated via a structured financial scheme when (1) the feedback survey (if the EMA report was discussed) and (2) at least 1 of the follow-up surveys are completed: €35 (approximately US $38) if compliance with the monitoring protocol is >33% (or >55 EMA surveys), €70 (approximately US $76) if compliance is >65% or (>109 EMA surveys), and €100 (approximately US $109) if compliance is >83% (or >139 EMA surveys). Otherwise, patients receive €20 (approximately US $22) in financial compensation. In addition, personalized feedback reports are provided so that relevant information can be fed back into the therapy room. Patients can also opt in to receive updates on the general findings of the project. Finally, mental health professionals receive €10 (approximately US $11) as compensation for completing the feedback survey after the clinical session in which they discuss the EMA feedback report. We do not provide a financial incentive for participation in the interviews (phase 3).

##### Research Training and Well-being

All research activities are performed by clinically trained research staff. Risk monitoring of people who frequently self-injure requires good-quality training in working with individuals who engage in NSSI and are at increased risk for suicide [19]. Therefore, research staff who interact with participants and are part of the risk-monitoring team are licensed clinical psychologists who have received additional training that covered how to support participants during an acute suicidal crisis. In addition, although there is a psychologist *on call* outside of office hours, responsibility is always shared with the first and second authors (GK: PhD level and good clinical practice certified and LC: PhD level and cognitive behavioral therapist), who are available for supervision by telephone during recruitment hours. Finally, there are regular debriefings to ensure the well-being of all team members throughout the study’s data collection period.

### Statistical Analyses

#### Overview

The psychometric properties of baseline measures (eg, Cronbach α), EMA multi-item scales (eg, multilevel reliability [120]), and interrater reliability of diagnostic interviews (a subset of 20% of the sample, approximately 24 participants) will be reported. To accommodate the hierarchical structure of the EMA data (eg, observations nested within individuals), our 3 primary aims will be analyzed by using a combination of statistical techniques. First, descriptive statistics (eg, means, modes, SDs, intraclass coefficients, and correlations) will be used to describe the sample in terms of baseline characteristics; compliance rates; presence; comorbidity patterns; and moment-to-moment variability of NSSI outcomes across minutes, hours, days, and weeks. Second, we will use dynamic structural equation modeling (DSEM), which integrates three modeling approaches [121,122]: (1) time series analysis, which allows for modeling the lagged relation between repeated measures in a single participant; (2) multilevel modeling, which takes the higher-order data structure into account and models these relationships for multiple participants while capturing variability within and between persons over time; and (3) structural equation modeling, which takes measurement error into account and allows for multiple outcome variables, latent variables, and mediation effects [123].

Using DSEM will clarify the extent to which momentary factors at time point *t*_–1_ (eg, negative affect) predict NSSI thoughts, NSSI urges, self-efficacy to resist NSSI, and NSSI behavior (or other self-damaging behaviors) at time point *t*, above and beyond the lagged version of the outcome variable (ie, the autoregressive parameter) and confounding variables at time point *t*_–1_ (eg, NSSI urges in the prediction of NSSI behavior). We will attempt to include random intercepts (predictors and outcomes) and random slopes (predictors) for momentary variables, with an unrestricted correlation structure if models converge (the maximum number of iterations is 50,000 by default). The time interval (Tinterval) statement will account for unequally spaced intervals owing to missing data and random sampling within blocks, with missing data handled using a Kalman filter approach [122]. Given that all participants have a recent history of NSSI but do not necessarily engage in other self-damaging behaviors, models that investigate within-person associations with disordered eating, substance use, and STB will be based on a subsample of participants that show variation in these secondary outcomes.

Third, we will use group iterative multiple model estimation (R package *gimme* [124]) to assess between-person variability in risk associations. Group iterative multiple model estimation estimates a unified structural equation model that includes lagged and contemporaneous relations in a network model [124,125] and accurately recovers group-, subgroup-, and individual-level associations in time series data. Fourth, fully idiographic modeling will be applied based on each participant’s time series data to identify the most relevant factors for each individual without overfitting models [126,127]. Elastic net regularization will be used as a statistical classification approach, which produces sparse models through coefficient penalization with k-fold cross-validation (R package *glmnet* [128]). Fifth, survival analysis will be used to investigate the factors that predict the transition from intense NSSI urges to behavior [129]. Importantly, however, as the analysis of intensive longitudinal data is a burgeoning field, newly available methods will also be considered (eg, continuous-time and Markov switching models [130-133]).

Finally, the secondary objective will be analyzed using a mixed methods approach. We will use thematic analysis to evaluate open-ended questions and interview data regarding end users’ experiences and perspectives [134]. Quantitative questionnaire data will be analyzed using descriptive statistics and regression analyses to explore associations with demographic and clinical characteristics.

#### Expected Sample Size

We intend to recruit 120 individuals seeking treatment. Analyses that include 100 to 120 participants will yield sufficient statistical power (≥0.80) to detect within-person effects as small as 0.07 to 0.09 in multilevel autoregressive models. These power calculations were conducted using Monte Carlo simulations with the following conservative estimates [135]: a 1:0.67 ratio of random intercept to within-person residual deviation, a 1:0.25 ratio of random intercept to random slope SD, first-order and slope-intercept correlations of 0.5, and compliance of 60% to the regular EMA surveys. Recent studies have used pooled machine learning models with fewer observations and participants [127]. In addition, it has been shown recently that as few as 60 assessments from 25 individuals allow robust estimation of general, shared, and person-specific temporal associations in intensive longitudinal data [136].

#### Open Science Statement

All materials of the DAILY project can be consulted on the project’s OSF page [115]. We will make the analysis plan, materials, and code of results in scientific publications available on the OSF page [137]. In addition, preprints will be uploaded on the PsyArXiv platform to ensure that findings are available to the wider research community. We do not have institutional review board approval to make the master data set publicly available, but we will make deidentified data available upon request for reproducibility purposes.

## Results

The study is funded by a postdoctoral fellowship from the Research Foundation Flanders (Belgium) awarded to the first author (June 2020: 12ZZM21N; Multimedia Appendix 1). The recruitment of participants began in June 2021 and data collection is anticipated to conclude in August 2023.

## Discussion

### Principal Findings

The DAILY project is expected to advance scientific knowledge of (1) the short-term course and within-person risk and protective factors of NSSI thoughts, NSSI urges, self-efficacy to resist NSSI, and NSSI behavior among individuals seeking treatment; (2) the transition from NSSI thoughts and urges to NSSI behavior; and (3) the comorbidity patterns, unique versus shared risk factors, and temporal relationships of NSSI with other self-damaging behaviors. Such information will inform clinical practice about how, why, and when self-injury and other self-damaging behaviors unfold in the everyday lives of individuals seeking treatment. Building upon these findings, we will explore the experiences and perspectives of end users (ie, people with lived experience and their mental health professionals) about the feasibility, scope, and utility of EMA and EMIs as digital clinical tools in treatment. This will provide the scientific building blocks for novel intervention approaches outside of the therapy room to support individuals who self-injure in real time. For instance, a sophisticated EMI that shows promise for dynamic behaviors such as NSSI and STB is just-in-time adaptive interventions [29,138,139]. This highly innovative intervention design adapts the provision of support in terms of type, intensity, and timing to an individual’s changing status and context to facilitate real-time interventions when and where they are needed most in everyday life and in ways that are appropriate and evidence based [140].

### Limitations

Although EMA is a powerful methodology that can now more readily be used to provide previously unavailable information about the course, within-person predictors, comorbidity patterns, and clinical outcomes of NSSI in daily life [19], it is crucial to consider the implications and limitations of the protocol used and the design when interpreting the project’s findings. First, although measures are in place to ensure that recruitment is inclusive and geographically representative, with recruitment sites spread across the entire Flanders region of Belgium, it should be acknowledged that we use convenience sampling, with the targeted sample comprising individuals currently receiving mental health treatment. This implies that the findings should not be generalized to nonclinical populations because the short-term course of NSSI will likely differ in severity for community samples. Relatedly, adolescents can only participate from the age of 15 years, meaning that the findings are not generalizable to early adolescents (aged 10-14 years) and should be studied in this population. Second, the intensive sampling scheme matches the project’s research objectives but involves a higher burden than traditional longitudinal studies (typically 1-5 surveys months to years apart) and most EMA studies (typically lasting 1 week) [26]. Although scholars have observed increased burden with longer questionnaires but not with increased sampling frequency among community samples [141], it should be investigated to what extent the current EMA sampling scheme and duration were considered feasible by all individuals seeking treatment. Third, we implemented safety measures for NSSI and other self-damaging behaviors (including the monitoring of suicide risk) based on prespecified cutoffs of momentary urges and low self-efficacy to resist a behavior. Future research would benefit from evaluating risk thresholds and the utility of different safety measures (eg, personal vs standard message and human-led vs automatic action) at varying risk levels for self-damaging behaviors.

Fourth, we operationalize NSSI and other self-damaging behaviors comprehensively (ie, thoughts, urges, and behavior) and assess a broad range of theoretically relevant factors (emotions, cognitions, contextual information, and social appraisals); however, as a consequence, most constructs rely on single EMA items that were selected from prior EMA studies or modified from conventional survey questionnaires to keep the workload for participants under control [141]. However, against the backdrop of a lack of psychometrically validated items for assessing psychological constructs with EMA [142] and a need to develop standardized measures [143], we will calculate the multilevel reliability of composite constructs (eg, negative affect) and made all EMA items (for the original Dutch items see the OSF page) publicly available [107]. Fifth, we provided additional (shorter) EMA surveys when participants report intense NSSI urges and contact participants and clinical staff when there is an increased suicide risk, which might result in underreporting of NSSI urges and STB. Sixth, because all participants receive mental health treatment, there might be structural changes in the descriptive patterns of self-damaging thoughts and behaviors across the monitoring period. We will evaluate this, and if the assumption of stationarity is violated, we will include days since study enrollment as a covariate for the outcome under investigation. Finally, we may miss moments leading up to self-damaging behaviors in the late evening and overnight as no EMA surveys are scheduled after 10 PM. Although we can assess to what extent this is the case for NSSI behavior (as people could register self-injury via the push button in the m-Path app), future research might benefit from sampling schemes adapted to people’s sleep and wake-up times.

### Conclusions

Notwithstanding the limitations, the findings of the DAILY project will provide a detailed characterization of the course and patterns of risk for NSSI during treatment by considering NSSI thoughts, NSSI urges, self-efficacy to resist NSSI, and NSSI behavior in the daily lives of individuals seeking treatment. This will help to increase our understanding of how NSSI unfolds across minutes, hours, days, and weeks. Filling these critical knowledge gaps using EMA will lay the foundation for, and guide, the development of novel interventions that support people when and where it matters most in daily life [29,138]. This scientific endeavor may facilitate young people’s recovery from self-injury and ultimately could help us prevent loss of life.

## Appendix

Multimedia Appendix 1 Peer review reports from Fonds voor Wetenschappelijk Onderzoek – Vlaanderen (FWO)/ Research Foundation Flanders (Belgium).

## Abbreviations

DAILY: Detection of Acute rIsk of seLf-injurY
DSEM: dynamic structural equation modeling
DSM-5: Diagnostic and Statistical Manual of Mental Disorders, Fifth Edition
EMA: ecological momentary assessment
EMI: ecological momentary intervention
GDPR: General Data Protection Regulation
NSSI: nonsuicidal self-injury
OSF: Open Science Framework
REDCap: Research Electronic Data Capture
STB: suicidal thoughts and behaviors

## Data Availability

Data sharing is not applicable to this paper as no data sets were analyzed.

## References

[1] Gillies D, Christou MA, Dixon AC, Featherston OJ, Rapti I, Garcia-Anguita A, Villasis-Keever M, Reebye P, Christou E, Al Kabir N, Christou PA (2018). Prevalence and characteristics of self-harm in adolescents: meta-analyses of community-based studies 1990-2015. J Am Acad Child Adolesc Psychiatry.

[2] Kiekens G, Hasking P, Bruffaerts R, Alonso J, Auerbach RP, Bantjes J, Benjet C, Boyes M, Chiu WT, Claes L, Cuijpers P, Ebert DD, Mak A, Mortier P, O'Neill S, Sampson NA, Stein DJ, Vilagut G, Nock MK, Kessler RC, WHO World Mental Health International College Student (WMH-ICS) collaborators (2023). Non-suicidal self-injury among first-year college students and its association with mental disorders: results from the world mental health international college student (WMH-ICS) initiative. Psychol Med.

[3] Gandhi A, Luyckx K, Baetens I, Kiekens G, Sleuwaegen E, Berens A, Maitra S, Claes L (2018). Age of onset of non-suicidal self-injury in Dutch-speaking adolescents and emerging adults: an event history analysis of pooled data. Compr Psychiatry.

[4] Steinhoff A, Ribeaud D, Kupferschmid S, Raible-Destan N, Quednow BB, Hepp U, Eisner M, Shanahan L (2021). Self-injury from early adolescence to early adulthood: age-related course, recurrence, and services use in males and females from the community. Eur Child Adolesc Psychiatry.

[5] Glenn CR, Lanzillo EC, Esposito EC, Santee AC, Nock MK, Auerbach RP (2017). Examining the course of suicidal and nonsuicidal self-injurious thoughts and behaviors in outpatient and inpatient adolescents. J Abnorm Child Psychol.

[6] Cucchi A, Ryan D, Konstantakopoulos G, Stroumpa S, Kaçar AŞ, Renshaw S, Landau S, Kravariti E (2016). Lifetime prevalence of non-suicidal self-injury in patients with eating disorders: a systematic review and meta-analysis. Psychol Med.

[7] Groschwitz RC, Kaess M, Fischer G, Ameis N, Schulze UM, Brunner R, Koelch M, Plener PL (2015). The association of non-suicidal self-injury and suicidal behavior according to DSM-5 in adolescent psychiatric inpatients. Psychiatry Res.

[8] Millon EM, Alqueza KL, Kamath RA, Marsh R, Pagliaccio D, Blumberg HP, Stewart JG, Auerbach RP (2022). Non-suicidal self-injurious thoughts and behaviors among adolescent inpatients. Child Psychiatry Hum Dev (forthcoming).

[9] Ose SO, Tveit T, Mehlum L (2021). Non-suicidal self-injury (NSSI) in adult psychiatric outpatients - a nationwide study. J Psychiatr Res.

[10] van Alphen NR, Stewart JG, Esposito EC, Pridgen B, Gold J, Auerbach RP (2017). Predictors of rehospitalization for depressed adolescents admitted to acute psychiatric treatment. J Clin Psychiatry.

[11] Burke TA, Piccirillo ML, Moore-Berg SL, Alloy LB, Heimberg RG (2019). The stigmatization of nonsuicidal self-injury. J Clin Psychol.

[12] Wilkinson PO, Qiu T, Neufeld S, Jones PB, Goodyer IM (2018). Sporadic and recurrent non-suicidal self-injury before age 14 and incident onset of psychiatric disorders by 17 years: prospective cohort study. Br J Psychiatry.

[13] Kiekens G, Claes L (2020). Non-suicidal self-injury and eating disordered behaviors: an update on what we do and do not know. Curr Psychiatry Rep.

[14] Goodman M, Tomas IA, Temes CM, Fitzmaurice GM, Aguirre BA, Zanarini MC (2017). Suicide attempts and self-injurious behaviours in adolescent and adult patients with borderline personality disorder. Personal Ment Health.

[15] Nock MK, Joiner Jr TE, Gordon KH, Lloyd-Richardson E, Prinstein MJ (2006). Non-suicidal self-injury among adolescents: diagnostic correlates and relation to suicide attempts. Psychiatry Res.

[16] Kiekens G, Hasking P, Boyes M, Claes L, Mortier P, Auerbach RP, Cuijpers P, Demyttenaere K, Green JG, Kessler RC, Myin-Germeys I, Nock MK, Bruffaerts R (2018). The associations between non-suicidal self-injury and first onset suicidal thoughts and behaviors. J Affect Disord.

[17] Ribeiro JD, Franklin JC, Fox KR, Bentley KH, Kleiman EM, Chang BP, Nock MK (2016). Self-injurious thoughts and behaviors as risk factors for future suicide ideation, attempts, and death: a meta-analysis of longitudinal studies. Psychol Med.

[18] Kiekens G, Claes L, Hasking P, Mortier P, Bootsma E, Boyes M, Myin-Germeys I, Demyttenaere K, Cuijpers P, Kessler RC, Nock MK, Bruffaerts R (2022). A longitudinal investigation of non-suicidal self-injury persistence patterns, risk factors, and clinical outcomes during the college period. Psychol Med (forthcoming).

[19] Kiekens G, Robinson K, Tatnell R, Kirtley OJ (2021). Opening the black box of daily life in nonsuicidal self-injury research: with great opportunity comes great responsibility. JMIR Ment Health.

[20] Franklin JC, Ribeiro JD, Fox KR, Bentley KH, Kleiman EM, Huang X, Musacchio KM, Jaroszewski AC, Chang BP, Nock MK (2017). Risk factors for suicidal thoughts and behaviors: a meta-analysis of 50 years of research. Psychol Bull.

[21] Fox KR, Franklin JC, Ribeiro JD, Kleiman EM, Bentley KH, Nock MK (2015). Meta-analysis of risk factors for nonsuicidal self-injury. Clin Psychol Rev.

[22] Kiekens G, Hasking P, Claes L, Mortier P, Auerbach RP, Boyes M, Cuijpers P, Demyttenaere K, Green JG, Kessler RC, Nock MK, Bruffaerts R (2018). The DSM-5 nonsuicidal self-injury disorder among incoming college students: prevalence and associations with 12-month mental disorders and suicidal thoughts and behaviors. Depress Anxiety.

[23] Kleiman EM, Nock MK (2018). Real-time assessment of suicidal thoughts and behaviors. Curr Opin Psychol.

[24] Myin-Germeys I, Kasanova Z, Vaessen T, Vachon H, Kirtley O, Viechtbauer W, Reininghaus U (2018). Experience sampling methodology in mental health research: new insights and technical developments. World Psychiatry.

[25] Shiffman S, Stone AA, Hufford MR (2008). Ecological momentary assessment. Annu Rev Clin Psychol.

[26] Myin-Germeys I, Kuppens P (2022). The Open Handbook of Experience Sampling Methodology: a Step-by-Step Guide to Designing, Conducting, and Analyzing ESM Studies. 2nd edition.

[27] Kiekens G, Hasking P, Nock MK, Boyes M, Kirtley O, Bruffaerts R, Myin-Germeys I, Claes L (2020). Fluctuations in affective states and self-efficacy to resist non-suicidal self-injury as real-time predictors of non-suicidal self-injurious thoughts and behaviors. Front Psychiatry.

[28] Kleiman EM, Bentley KH, Glenn CR, Liu RT, Rizvi SL (2022). Building on the past 50 years, not starting over: a balanced interpretation of meta-analyses, reviews, and commentaries on treatments for suicide and self-injury. Gen Hosp Psychiatry.

[29] Coppersmith DD, Dempsey W, Kleiman EM, Bentley KH, Murphy SA, Nock MK (2022). Just-in-time adaptive interventions for suicide prevention: promise, challenges, and future directions. Psychiatry.

[30] Nock MK, Prinstein MJ, Sterba SK (2009). Revealing the form and function of self-injurious thoughts and behaviors: a real-time ecological assessment study among adolescents and young adults. J Abnorm Psychol.

[31] Fitzpatrick S, Kranzler A, Fehling K, Lindqvist J, Selby EA (2020). Investigating the role of the intensity and duration of self-injury thoughts in self-injury with ecological momentary assessment. Psychiatry Res.

[32] Turner BJ, Baglole JS, Chapman AL, Gratz KL (2019). Experiencing and resisting nonsuicidal self-injury thoughts and urges in everyday life. Suicide Life Threat Behav.

[33] Hasking P, Whitlock J, Voon D, Rose A (2017). A cognitive-emotional model of NSSI: using emotion regulation and cognitive processes to explain why people self-injure. Cogn Emot.

[34] Hooley JM, Franklin JC (2018). Why do people hurt themselves? A new conceptual model of nonsuicidal self-injury. Clin Psychol Sci.

[35] Selby EA, Anestis MD, Joiner TE (2008). Understanding the relationship between emotional and behavioral dysregulation: emotional cascades. Behav Res Ther.

[36] Rodríguez-Blanco L, Carballo JJ, Baca-García E (2018). Use of ecological momentary assessment (EMA) in non-suicidal self-injury (NSSI): a systematic review. Psychiatry Res.

[37] Gee BL, Han J, Benassi H, Batterham PJ (2020). Suicidal thoughts, suicidal behaviours and self-harm in daily life: a systematic review of ecological momentary assessment studies. Digit Health.

[38] Hepp J, Carpenter RW, Störkel LM, Schmitz SE, Schmahl C, Niedtfeld I (2020). A systematic review of daily life studies on non-suicidal self-injury based on the four-function model. Clin Psychol Rev.

[39] Victor SE, Scott LN, Stepp SD, Goldstein TR (2019). I want you to want me: interpersonal stress and affective experiences as within-person predictors of nonsuicidal self-injury and suicide urges in daily life. Suicide Life Threat Behav.

[40] Turner BJ, Yiu A, Claes L, Muehlenkamp JJ, Chapman AL (2016). Occurrence and co-occurrence of nonsuicidal self-injury and disordered eating in a daily diary study: which behavior, when?. Psychiatry Res.

[41] Brown AC, Dhingra K, Brown TD, Danquah AN, Taylor PJ (2022). A systematic review of the relationship between momentary emotional states and nonsuicidal self-injurious thoughts and behaviours. Psychol Psychother.

[42] Miller AB, Eisenlohr-Moul T, Glenn CR, Turner BJ, Chapman AL, Nock MK, Prinstein MJ (2019). Does higher-than-usual stress predict nonsuicidal self-injury? Evidence from two prospective studies in adolescent and emerging adult females. J Child Psychol Psychiatry.

[43] Burke TA, Fox K, Kautz M, Siegel DM, Kleiman E, Alloy LB (2021). Real-time monitoring of the associations between self-critical and self-punishment cognitions and nonsuicidal self-injury. Behav Res Ther.

[44] Hughes CD, King AM, Kranzler A, Fehling K, Miller A, Lindqvist J, Selby EA (2019). Anxious and overwhelming affects and repetitive negative thinking as ecological predictors of self-injurious thoughts and behaviors. Cogn Ther Res.

[45] Wright AG, Woods WC (2020). Personalized models of psychopathology. Annu Rev Clin Psychol.

[46] Kaurin A, Dombrovski AY, Hallquist MN, Wright AG (2022). Integrating a functional view on suicide risk into idiographic statistical models. Behav Res Ther.

[47] Klonsky ED, Saffer BY, Bryan CJ (2018). Ideation-to-action theories of suicide: a conceptual and empirical update. Curr Opin Psychol.

[48] Fox KR, Wang SB, Boccagno C, Haynos AF, Kleiman E, Hooley JM (2019). Comparing self-harming intentions underlying eating disordered behaviors and NSSI: evidence that distinctions are less clear than assumed. Int J Eat Disord.

[49] Kleiman EM, Coppersmith DD, Millner AJ, Franz PJ, Fox KR, Nock MK (2018). Are suicidal thoughts reinforcing? A preliminary real-time monitoring study on the potential affect regulation function of suicidal thinking. J Affect Disord.

[50] Cho SB, Su J, Kuo SI, Bucholz KK, Chan G, Edenberg HJ, McCutcheon VV, Schuckit MA, Kramer JR, Dick DM (2019). Positive and negative reinforcement are differentially associated with alcohol consumption as a function of alcohol dependence. Psychol Addict Behav.

[51] Wycoff AM, Metrik J, Trull TJ (2018). Affect and cannabis use in daily life: a review and recommendations for future research. Drug Alcohol Depend.

[52] Czyz EK, Glenn CR, Busby D, King CA (2019). Daily patterns in nonsuicidal self-injury and coping among recently hospitalized youth at risk for suicide. Psychiatry Res.

[53] Czyz EK, Glenn CR, Arango A, Koo HJ, King CA (2021). Short-term associations between nonsuicidal and suicidal thoughts and behaviors: a daily diary study with high-risk adolescents. J Affect Disord.

[54] Robillard CL, Legg NK, Ames ME, Turner BJ (2022). Support for a transdiagnostic motivational model of self-damaging behaviors: comparing the salience of motives for binge drinking, disordered eating, and nonsuicidal self-injury. Behav Ther.

[55] Kleiman EM, Turner BJ, Fedor S, Beale EE, Picard RW, Huffman JC, Nock MK (2018). Digital phenotyping of suicidal thoughts. Depress Anxiety.

[56] Bos FM, Snippe E, Bruggeman R, Wichers M, van der Krieke L (2019). Insights of patients and clinicians on the promise of the experience sampling method for psychiatric care. Psychiatr Serv.

[57] Weermeijer J, Kiekens G, Wampers M, Kuppens P, Myin-Germeys IM (2023). Practitioner perspectives on the use of the experience sampling software in counseling and clinical psychology. Behav Inf Technol (forthcoming).

[58] Kivelä L, van der Does WA, Riese H, Antypa N (2022). Don't miss the moment: a systematic review of ecological momentary assessment in suicide research. Front Digit Health.

[59] Gromatsky M, Patel TA, Wilson SM, Mann AJ, Aho N, Carpenter VL, Calhoun PS, Beckham JC, Goodman M, Kimbrel NA (2022). Qualitative analysis of participant experiences during an ecological momentary assessment study of nonsuicidal self-injury among veterans. Psychiatry Res.

[60] Arshad U, Gauntlett J, Husain N, Chaudhry N, Taylor PJ, Farhat-Ul-Ain (2020). A systematic review of the evidence supporting mobile- and internet-based psychological interventions for self-harm. Suicide Life Threat Behav.

[61] Kruzan KP, Whitlock J, Chapman J, Bhandari A, Bazarova N (2023). Young adults' perceptions of 2 publicly available digital resources for self-injury: qualitative study of a peer support app and web-based factsheets. JMIR Form Res.

[62] Kruzan KP, Meyerhoff J, Biernesser C, Goldstein T, Reddy M, Mohr DC (2021). Centering lived experience in developing digital interventions for suicide and self-injurious behaviors: user-centered design approach. JMIR Ment Health.

[63] Harris PA, Taylor R, Minor BL, Elliott V, Fernandez M, O'Neal L, McLeod L, Delacqua G, Delacqua F, Kirby J, Duda SN, REDCap Consortium (2019). The REDCap consortium: building an international community of software platform partners. J Biomed Inform.

[64] Nock MK, Holmberg EB, Photos VI, Michel BD (2007). Self-injurious thoughts and behaviors interview: development, reliability, and validity in an adolescent sample. Psychol Assess.

[65] Fox KR, Harris JA, Wang SB, Millner AJ, Deming CA, Nock MK (2020). Self-injurious thoughts and behaviors interview-revised: development, reliability, and validity. Psychol Assess.

[66] First MB, Williams JB, Karg RS, Spitzer RL (2016). User's Guide for the SCID-5-CV Structured Clinical Interview for DSM-5® Disorders: Clinical Version.

[67] Landis JR, Koch GG (1977). An application of hierarchical kappa-type statistics in the assessment of majority agreement among multiple observers. Biometrics.

[68] Mestdagh M, Verdonck S, Piot M, Niemerijer K, Tuerlinckx F, Kuppens P, Dejonckheere E (2022). m-Path: an easy-to-use and flexible platform for ecological momentary assessment and intervention in behavioral research and clinical practice. PsyArXiv.

[69] Watson D, Clark LA, Tellegen A (1988). Development and validation of brief measures of positive and negative affect: the PANAS scales. J Pers Soc Psychol.

[70] Engelen U, De Peuter S, Victoir A, Van Diest I, Van den Bergh O (2006). Verdere validering van de Positive and Negative Affect Schedule (PANAS) en vergelijking van twee Nederlandstalige versies. Gedrag Gezond.

[71] Treynor W, Gonzalez R, Nolen-Hoeksema S (2003). Rumination reconsidered: a psychometric analysis. Cognit Ther Res.

[72] Raes F, Schoofs H, Hoes D, Hermans D, van den Eede F, Franck E (2009). 'Reflection' en 'brooding' als subtypes van rumineren: Een herziening van de Ruminative Response Scale. Gedragstherapie.

[73] Ehring T, Zetsche U, Weidacker K, Wahl K, Schönfeld S, Ehlers A (2011). The perseverative thinking questionnaire (PTQ): validation of a content-independent measure of repetitive negative thinking. J Behav Ther Exp Psychiatry.

[74] Ehring T, Raes F, Weidacker K, Emmelkamp PM (2012). Validation of the Dutch version of the perseverative thinking questionnaire (PTQ-NL). Eur J Psychol Assess.

[75] Gilbert P, Clarke M, Hempel S, Miles JN, Irons C (2004). Criticizing and reassuring oneself: an exploration of forms, styles and reasons in female students. Br J Clin Psychol.

[76] Sommers-Spijkerman M, Trompetter H, Ten Klooster P, Schreurs K, Gilbert P, Bohlmeijer E (2018). Development and validation of the forms of self-criticizing/attacking and self-reassuring scale-short form. Psychol Assess.

[77] Kaufman EA, Xia M, Fosco G, Yaptangco M, Skidmore CR, Crowell SE (2015). The difficulties in emotion regulation scale short form (DERS-SF): validation and replication in adolescent and adult samples. J Psychopathol Behav Assess.

[78] Neumann A, van Lier PA, Gratz KL, Koot HM (2010). Multidimensional assessment of emotion regulation difficulties in adolescents using the difficulties in emotion regulation scale. Assessment.

[79] Cyders MA, Littlefield AK, Coffey S, Karyadi KA (2014). Examination of a short English version of the UPPS-P impulsive behavior scale. Addict Behav.

[80] Claes L, Muehlenkamp J (2013). The relationship between the UPPS-P impulsivity dimensions and nonsuicidal self-injury characteristics in male and female high-school students. Psychiatry J.

[81] Rosenthal DA, Gurney RM, Moore SM (1981). From trust on intimacy: a new inventory for examining Erikson's stages of psychosocial development. J Youth Adolesc.

[82] Claes L, Luyckx K, Bijttebier P (2014). Non-suicidal self-injury in adolescents: prevalence and associations with identity formation above and beyond depression. Pers Individ Dif.

[83] Henry JD, Crawford JR (2005). The short-form version of the Depression Anxiety Stress Scales (DASS-21): construct validity and normative data in a large non-clinical sample. Br J Clin Psychol.

[84] de Beurs E, Van Dyck R, Marquenie LA, Lange A, Blonk RW (2001). De DASS: Een vragenlijst voor het meten van depressie, angst en stress. Gedragstherapie.

[85] Hasking P, Boyes M (2018). The non-suicidal self-injury expectancy questionnaire: factor structure and initial validation. Clin Psychol.

[86] Czyz EK, Bohnert AS, King CA, Price AM, Kleinberg F, Ilgen MA (2014). Self-efficacy to avoid suicidal action: factor structure and convergent validity among adults in substance use disorder treatment. Suicide Life Threat Behav.

[87] Dawkins JC, Hasking PA, Boyes ME (2021). Development and validation of a measure of self-efficacy to resist nonsuicidal self-injury. J Psychopathol Behav Assess.

[88] Fairburn CG, Beglin SJ, Fairburn CG (2008). Eating disorder examination questionnaire (EDE-Q 6.0). Cognitive Behavior Therapy and Eating Disorders.

[89] Aardoom JJ, Dingemans AE, Fokkema M, Spinhoven P, Van Furth EF (2017). Moderators of change in an internet-based intervention for eating disorders with different levels of therapist support: what works for whom?. Behav Res Ther.

[90] Tylka TL, Wood-Barcalow NL (2015). The body appreciation scale-2: item refinement and psychometric evaluation. Body Image.

[91] Palmeroni N Perfection hurts: identity formation and body image throughout adolescence and emerging adulthood. Faculty of Psychology and Educational Sciences, KU Leuven.

[92] Hohlstein LA, Smith GT, Atlas JG (1998). An application of expectancy theory to eating disorders: development and validation of measures of eating and dieting expectancies. Psychol Assess.

[93] Dingemans AE, Martijn C, van Furth EF, Jansen AT (2009). Expectations, mood, and eating behavior in binge eating disorder. Beware of the bright side. Appetite.

[94] Marinilli Pinto A, Guarda AS, Heinberg LJ, Diclemente CC (2006). Development of the eating disorder recovery self-efficacy questionnaire. Int J Eat Disord.

[95] Zanarini MC, Vujanovic AA, Parachini EA, Boulanger JL, Frankenburg FR, Hennen J (2003). A screening measure for BPD: the McLean screening instrument for borderline personality disorder (MSI-BPD). J Pers Disord.

[96] Verschuere B, Tibboel H (2011). De Nederlandstalige versie van de McLean Screening Instrument for borderline personality disorder (MSI-BPD). Psychol Gezondh.

[97] Oei TP, Hasking PA, Young RM (2005). Drinking refusal self-efficacy questionnaire-revised (DRSEQ-R): a new factor structure with confirmatory factor analysis. Drug Alcohol Depend.

[98] Voogt CV, Kuntsche E, Kleinjan M, Engels RC (2014). The effect of the 'what do you drink' web-based brief alcohol intervention on self-efficacy to better understand changes in alcohol use over time: randomized controlled trial using ecological momentary assessment. Drug Alcohol Depend.

[99] Jerusalem M, Schwarzer R, Schwarzer R (1992). Self-efficacy as a resource factor in stress appraisal processes. Self-Efficacy: Thought Control of Action.

[100] Teeuw B, Schwarzer R, Jerusalem M (1994). Dutch general self-efficacy scale. Central Facility for Data Processing (ZEDAT).

[101] Zimet GD, Dahlem NW, Zimet SG, Farley GK (1988). The multidimensional scale of perceived social support. J Pers Assess.

[102] Pedersen SS, Spinder H, Erdman RA, Denollet J (2009). Poor perceived social support in implantable cardioverter defibrillator (ICD) patients and their partners: cross-validation of the multidimensional scale of perceived social support. Psychosomatics.

[103] Cyranowski JM, Zill N, Bode R, Butt Z, Kelly MA, Pilkonis PA, Salsman JM, Cella D (2013). Assessing social support, companionship, and distress: National Institute of Health (NIH) toolbox adult social relationship scales. Health Psychol.

[104] Edison SW, Geissler GL (2003). Measuring attitudes towards general technology: antecedents, hypotheses and scale development. J Target Meas Anal Mark.

[105] Arntz A, Kamphuis JH, Derks JL (2017). SCID-5-S: Gestructureerd klinisch interview voor DSM-5 Syndroomstoornissen.

[106] Kiekens G, Hasking P, Nock MK, Kleiman E, Kirtley O, Houben M, Boyes M, Bruffaerts R, Myin-Germeys I, Claes L (2022). A comparison of affective-cognitive dynamics in daily life between emerging adults with and without past-year non-suicidal self-injury. PsyArXiv.

[107] Kirtley O, Hiekkaranta AP, Kunkels YK, Eisele G, Schoefs S, Kemme N, Le Grange J, Simsa B, Biesemans T, Van Heck L, Myin-Germeys I (2018). The experience sampling method (ESM) item repository. Open Science Framework.

[108] Moberly NJ, Watkins ER (2008). Ruminative self-focus, negative life events, and negative affect. Behav Res Ther.

[109] Russell JA (2003). Core affect and the psychological construction of emotion. Psychol Rev.

[110] Blades CA, Stritzke WG, Page AC, Brown JD (2018). The benefits and risks of asking research participants about suicide: a meta-analysis of the impact of exposure to suicide-related content. Clin Psychol Rev.

[111] Hasking P, Tatnell RC, Martin G (2015). Adolescents' reactions to participating in ethically sensitive research: a prospective self-report study. Child Adolesc Psychiatry Ment Health.

[112] Coppersmith DD, Fortgang RG, Kleiman EM, Millner AJ, Yeager AL, Mair P, Nock MK (2022). Effect of frequent assessment of suicidal thinking on its incidence and severity: high-resolution real-time monitoring study. Br J Psychiatry.

[113] Nock MK, Kleiman EM, Abraham M, Bentley KH, Brent DA, Buonopane RJ, Castro-Ramirez F, Cha CB, Dempsey W, Draper J, Glenn CR, Harkavy-Friedman J, Hollander MR, Huffman JC, Lee HI, Millner AJ, Mou D, Onnela JP, Picard RW, Quay HM, Rankin O, Sewards S, Torous J, Wheelis J, Whiteside U, Siegel G, Ordóñez AE, Pearson JL (2021). Consensus statement on ethical and safety practices for conducting digital monitoring studies with people at risk of suicide and related behaviors. Psychiatr Res Clin Pract.

[114] Bai S, Babeva KN, Kim MI, Asarnow JR (2021). Future directions for optimizing clinical science and safety: ecological momentary assessments in suicide/self-harm research. J Clin Child Adolesc Psychol.

[115] Center for Open Science.

[116] Grant AM, Franklin J, Langford P (2002). The self-reflection and insight scale: a new measure of private self-consciousness. Soc Behav Pers.

[117] Hatcher RL, Gillaspy JA (2006). Development and validation of a revised short version of the working alliance inventory. Psychother Res.

[118] Llewelyn SP (1988). Psychological therapy as viewed by clients and therapists. Br J Clin Psychol.

[119] Stinckens N, Geys K, Vos E, Vrancken M, Smits D, Claes L (2015). Wat cliënten ons te vertellen hebben over psychotherapie: Een kwalitatief onderzoek naar veranderingen en veranderingsmechanismen. Tijdschr Psychiatr.

[120] Shrout PE, Lane SP, Cooper H, Camic PM, Long DL, Panter AT, Rindskopf D, Sher KJ (2012). Reliability. APA Handbook of Research Methods in Psychology, Volume 1: Foundations, Planning, Measures, and Psychometrics.

[121] Hamaker EL, Asparouhov T, Muthén B, Hoyle RH (2022). Dynamic structural equation modeling as a combination of time series modeling, multilevel modeling, structural equation modeling. Handbook of Structural Equation Modeling. 2nd edition.

[122] McNeish D, Hamaker EL (2020). A primer on two-level dynamic structural equation models for intensive longitudinal data in Mplus. Psychol Methods.

[123] McNeish D, MacKinnon DP (2022). Intensive longitudinal mediation in Mplus. Psychol Methods (forthcoming).

[124] Lane ST, Gates KM (2017). Automated selection of robust individual-level structural equation models for time series data. Struct Equ Modeling.

[125] Beltz AM, Gates KM (2017). Network mapping with GIMME. Multivariate Behav Res.

[126] Fisher AJ, Soyster P (2019). Generating accurate personalized predictions of future behavior: a smoking exemplar. PsyArXiv.

[127] Soyster PD, Ashlock L, Fisher AJ (2022). Pooled and person-specific machine learning models for predicting future alcohol consumption, craving, and wanting to drink: a demonstration of parallel utility. Psychol Addict Behav.

[128] Hastie T, Qian J, Tay K (2022). An introduction to glmnet. The Comprehensive R Archive Network.

[129] Therneau TM (2022). Mixed effects cox models. The Comprehensive R Archive Network.

[130] Golino H, Nesselroade J, Christensen AP (2022). Towards a psychology of individuals: the ergodicity information index and a bottom-up approach for finding generalizations. PsyArXiv.

[131] Driver CC, Oud JH, Voelkle MC (2017). Continuous time structural equation modeling with R package ctsem. J Stat Softw.

[132] Koslovsky MD, Hébert ET, Businelle MS, Vannucci M (2020). A Bayesian time-varying effect model for behavioral mHealth data. Ann Appl Stat.

[133] Koslovsky MD, Swartz MD, Chan W, Leon-Novelo L, Wilkinson AV, Kendzor DE, Businelle MS (2018). Bayesian variable selection for multistate Markov models with interval-censored data in an ecological momentary assessment study of smoking cessation. Biometrics.

[134] Braun V, Clarke V (2022). Thematic Analysis: A Practical Guide.

[135] Lafit G, Adolf JK, Dejonckheere E, Myin-Germeys I, Viechtbauer W, Ceulemans E (2021). Selection of the number of participants in intensive longitudinal studies: a user-friendly shiny app and tutorial for performing power analysis in multilevel regression models that account for temporal dependencies. Adv Methods Pract Psychol Sci.

[136] Lane ST, Gates KM, Pike HK, Beltz AM, Wright AG (2019). Uncovering general, shared, and unique temporal patterns in ambulatory assessment data. Psychol Methods.

[137] Kirtley OJ, Janssens JJ, Kaurin A (2022). Open science in suicide research is open for business. Crisis.

[138] Ghahramanlou-Holloway M (2022). Entering the brave new world of just-in-time adaptive interventions for suicide prevention. Psychiatry.

[139] Wang L, Miller LC (2020). Just-in-the-moment adaptive interventions (JITAI): a meta-analytical review. Health Commun.

[140] Nahum-Shani I, Smith SN, Spring BJ, Collins LM, Witkiewitz K, Tewari A, Murphy SA (2018). Just-in-time adaptive interventions (JITAIs) in mobile health: key components and design principles for ongoing health behavior support. Ann Behav Med.

[141] Eisele G, Vachon H, Lafit G, Kuppens P, Houben M, Myin-Germeys I, Viechtbauer W (2022). The effects of sampling frequency and questionnaire length on perceived burden, compliance, and careless responding in experience sampling data in a student population. Assessment.

[142] Wright AG, Zimmermann J (2019). Applied ambulatory assessment: integrating idiographic and nomothetic principles of measurement. Psychol Assess.

[143] Mestdagh M, Dejonckheere E (2021). Ambulatory assessment in psychopathology research: current achievements and future ambitions. Curr Opin Psychol.

