# Supplementary material for: The Detection of Acute Risk of Self-injury Project: Protocol for an Ecological Momentary Assessment Study Among Individuals Seeking Treatment
Source: JMIR Res Protoc. 2023 Jun 15;12:e46244. doi: 10.2196/46244 (PMC10337382; doi:10.2196/46244)
Supplement: Multimedia Appendix 1 [file resprot_v12i1e46244_app1.pdf]

fwo
Opening
new
horizons

Applicant: Glenn Kiekens
Application number: 12ZZM21N
24 juni 2020

Concerning Evaluation application Postdoctoral Fellow - junior: ”The DAILY project: Detection of Acute rIsk of seLf-injurY in real-life.”

Dear researcher,

This year, the FWO received 703 applications for a Postdoctoral Fellow - junior, 181 of which could eventually be granted by the board of trustees after a rigorous selection procedure.

The expert panel G&M3PD: Psychology, Pedagogy, Didactics and Social Work has carefully evaluated your application. Based on the final ranking of the candidates in this panel, your application was selected for granting.

Consequently, the board of trustees has appointed you as postdoc fellow.

Attached you can find detailed feedback on the evaluation. As a reference to the comments and scores, we refer to the score grid: https://www.fwo.be/media/1023749/postdoc_preselection-scoring-grids-v2020.pdf

There will be no further correspondence about this feedback.

With kind regards,

your account administrator

fwo
Opening
new
horizons

Applicant: Glenn Kiekens
Application number: 12ZZM21N
24 juni 2020

Assessment criterion “candidate”

Feedback

This is a good candidate with strong publications, two masters degrees, and some awards.

The motivation of the candidate can be classified as high. His previous publishing activities are exactly in the field of the proposal now submitted and thus represent a continuation of skills and knowledge already acquired. Furthermore, the candidate has an excellent network of supporters.

The candidate has made significant contributions to the field of study of NSSI to date. This has included first-author publications in some mid- to high-impact journals. He has worked closely (and published with) recognized NSSI experts in the United States as well as in his local Universities. His training has included an advanced degree in statistics as well as Ph.D.’s in both psychology and public health. He has brought in two high paying grants/awards, at least one of which had an international call for applications. He has had a number of smaller awards as well.

There is clearly evidence of emerging international scientific reputation with a clear upward trajectory for the candidate. The candidate has meaningful contributions to the state of the art, which are properly acknowledged in the scientific community in the form of scholarly peer-reviewed publications. Further, there is impact beyond publications, such as being included as an early-career collaborator with an Austrian group for trigger evaluation of non-suicidal self-injury in online networks, and being invited to Vienna to develop a curriculum for school-based prevention for non-suicidal self-injury. The candidate is developing his scientific independence.

Score: 6,25

fwo
Opening
new
horizons

Applicant: Glenn Kiekens
Application number: 12ZZM21N
24 juni 2020

Assessment criterion “project”

Feedback

The project aims to examine daily predictors of non-suicidal self-injury in 100 individuals who self-injure. The description is clear and the aims are important. Three objectives will be pursued by analysing the same data set. Some of the research gaps identified in the proposal seem somewhat surprising; is there really no research examining the factors that increase the transition from NSSI thoughts to behaviour? All in all, while the project is worthwhile, one is not sure it is sufficient in volume, particularly when one compares it to other applications.

Comments made by evaluators include:

- The most important issue of this project concerns its feasibility. One feels that two of the three objectives would already be sufficient for a three-year project.

One wonders whether there will be enough observations per participant to run the participant-specific models. Assuming a response rate of 70%, you would have 30*8*.7 = 168 data points, of which you will use 126 for development of the model. A fear is that this is not enough data to get a stable model.

- One felt that the incorporation of passive monitoring data is a long shot. The candidate also relies om someone else to do this.

One fully agrees with the research gaps 1-3. These are important issues that the research community needs to address. A very high priority seems to be an individualized, ideographic approach, where risk stratification for NSSI takes place at the level of individuals.

Predicting the acute risk of NSSI thinking and behavior requires a larger number of observations that would need to be evaluated more closely (in hours, days, weeks and months).

Objectives: The mobile phone app does not guarantee secure input (keyword: compliance), especially for potentially suicidal patients? The applicant reports in his own publications with his co-authors that 20% are non-compliant: is there already more information on this - are these patients with severe symptoms who would need monitoring most urgently (keyword: prevention)? These questions also apply to other studies with a compliance rate of 62-83%.

Shouldn't this also be systematically checked in DAILY, i.e. i) compliance and ii) percentage participation according to health condition/diagnosis (also according to RDoC)?

A further question would be whether a compliance-independent physiological factor, such as actimetry, should not be additionally collected, which could provide additional information for a prediction.

Minor: Please provide a source for the mobile app - is it open source?

The research proposal to investigate the non-suicidal self-injury (NSSI) of young people more intensively is of high clinical relevance. Longitudinal studies of within-subject data over short periods of time over a longer period of time are a very promising approach.

The DAILY project submitted here is excellently planned and the applicant meets all criteria for a successful implementation of this innovative proposal.

This proposal raises some very interesting and important questions. It is noteworthy that the applicant raises the problem of ergodicity – or the fact that what is true of a group is not necessarily true of individuals within a group. The reality of the ecological fallacy is the main rationale for conducting this research. While we have considerable research on the characteristics of groups of individuals who engage in NSSI, we really know very little about individuals who engage in NSSI over time. The main concern of NSSI is hospital time/cost and risk of suicide.

The first objective relates to developing an individual prediction model for detecting NSSI thoughts and behaviors. The researcher will look at emotional factors, situational factors and some cognitive factors.

There is brief mention of a physiological variable in the form of galvanic skin response, which will be collected using a wearable wireless band. Later in the application it is clarified that the wearable wristband also collects skin temperature, heart rate variability and motion. It is unclear why there is not more emphasis on biological/physiological variables as predictors of behavior and/or thoughts of NSSI.

The advantage that physiological variables have is that they are much more objective/reliably measured than emotional or cognitive factors. Additionally, unlike the others, which involve discrete time-points during which data are collected, with large intervals during which data are not collected, physiological variables can be collected continuously. Since the brain is the "final common pathway" for physiology, emotion, cognition and behavior, it seems logical to posit a biological predictor of NSSI thoughts and behaviors and suicidal acts. Prior research has indicated that habituation to aversive stimuli is abnormal in women with NSSI, for instance. One of the collaborators on this project (M. Nock), has published about respiratory sinus arrhythmia differences associated with suicidal ideation. Yet here are no hypotheses about physiological variables. If the research team is going to all the effort to conduct this study and collect physiological data one strongly recommends including that component in the objectives, with a hypothesis.

Noteworthy is that 3 of 5 locations from which recruitment will take place are eating disorder units. Eating disorders frequently affect physiological variables and this is another reason why it will be important to evaluate them. There may be differences in NSSI among patients with different physiological profiles.

Objective 2 suffers from the same absence of physiological awareness as objective 1. It is easy to justify that transition from NSSI thoughts to actions would be accompanied by physiological changes. Why not make a hypothesis and test it?

Regarding Objective 3, the ecological fallacy makes it particularly relevant to understand if the association between NSSI and suicide attempts that has been found in groups of research subjects is applicable within individuals. Patients who engage in NSSI sometimes communicate that they do it to prevent an act of suicide. Thus, it is not a foregone conclusion that NSSI predicts suicide attempts for any given person. This goal is perhaps the most important in the proposal because it involves the most serious risk to individuals in the NSSI population.

fwo
Opening
new
horizons

Even with 100 patients who engage in NSSI, the rate of suicide attempts over 1-month interval is likely to be low. This could create challenges in predictions or associations within the analyses. This is worth considering as a potential risk to the study (specifically, objective 3). For example, if there are only 3 suicide attempts within the subjects in the time-frame of study, will that really be enough to determine any individual predictors? The candidate has determined the number of potential participants meeting inclusion criteria at each site from extant data. Can he also estimate rates of suicide attempts in these populations in a 1-month time interval? These patients are in psychiatric care (or recently discharged from inpatient care) so treatment will be active, hopefully reducing suicide attempts (at least for those recruited from outpatient clinics who are likely to be less acutely ill).

It would be worth considering how generalizable the results of this study will be given the very specific population of patients with quite high levels of NSSI. It makes sense to use this population so that there are some data points for NSSI, but as a consequence, the results may not pertain to patients who engage in NSSI on a once daily, weekly, or much less frequent basis.

The candidate mentioned that "…participants will be required to register responses within 10 minutes." Would this mean, operationally, that any data entered after 10 minutes will not be used? Or perhaps will not even be recorded?

score: 5,5
